# Predictive models for short-term mortality and length of hospital stay among adults with community-onset bacteraemia before and during the COVID-19 pandemic: application of early data dynamics

**DOI:** 10.1186/s12879-023-08547-8

**Published:** 2023-09-15

**Authors:** Ching-Chi Lee, Yuan-Pin Hung, Chih-Chia Hsieh, Ching-Yu Ho, Chiao-Ya Hsu, Cheng-Te Li, Wen-Chien Ko

**Affiliations:** 1 Clinical Medical Research Center, College of Medicine, National Cheng Kung University Hospital, National Cheng Kung University, Tainan, Taiwan; 2 Department of Internal Medicine, College of Medicine, National Cheng Kung University Hospital, National Cheng Kung University, No. 138, Sheng Li Road, Tainan, 70403 Taiwan; 3 Department of Internal Medicine, Tainan Hospital, Ministry of Health and Welfare, Tainan, Taiwan; 4 Department of Medicine, College of Medicine, National Cheng Kung University, Tainan, Taiwan; 5 Department of Emergency Medicine, College of Medicine, National Cheng Kung University Hospital, National Cheng Kung University, Tainan, Taiwan; 6 Department of Adult Critical Care Medicine, Tainan Sin-Lau Hospital, Tainan, Taiwan; 7 Department of Nursing, National Tainan Junior College of Nursing, Tainan, Taiwan; 8 Institute of Data Science, National Cheng Kung University, No. 1, University Road, Tainan, 701 Taiwan

**Keywords:** Prediction model, Community-onset, Bloodstream infections, Length of hospital stay, Mortality, COVID-19

## Abstract

**Background:**

The development of scoring systems to predict the short-term mortality and the length of hospital stay (LOS) in patients with bacteraemia is essential to improve the quality of care and reduce the occupancy variance in the hospital bed.

**Methods:**

Adults hospitalised with community-onset bacteraemia in the coronavirus disease 2019 (COVID-19) and pre-COVID-19 eras were captured as the validation and derivation cohorts in the multicentre study, respectively. Model I incorporated all variables available on day 0, Model II incorporated all variables available on day 3, and Models III, IV, and V incorporated the variables that changed from day 0 to day 3. This study adopted the statistical and machine learning (ML) methods to jointly determine the prediction performance of these models in two study cohorts.

**Results:**

A total of 3,639 (81.4%) and 834 (18.6%) patients were included in the derivation and validation cohorts, respectively. Model IV achieved the best performance in predicting 30-day mortality in both cohorts. The most frequently identified variables incorporated into Model IV were deteriorated consciousness from day 0 to day 3 and deteriorated respiration from day 0 to day 3. Model V achieved the best performance in predicting LOS in both cohorts. The most frequently identified variables in Model V were deteriorated consciousness from day 0 to day 3, a body temperature ≤ 36.0 °C or ≥ 39.0 °C on day 3, and a diagnosis of complicated bacteraemia.

**Conclusions:**

For hospitalised adults with community-onset bacteraemia, clinical variables that dynamically changed from day 0 to day 3 were crucial in predicting the short-term mortality and LOS.

**Supplementary Information:**

The online version contains supplementary material available at 10.1186/s12879-023-08547-8.

## Introduction

Despite recent advancements in haemodynamic support and antimicrobial strategies, bacteraemia remains strongly associated with high morbidity and mortality, leading to substantial healthcare costs [1]. Bacteraemia is a complex infection with varied clinical presentations and mortality rates, depending on the severity of the illness, the patient’s immune status, comorbid severity, causative microorganisms, and infection sources [2, 3]. Therefore, several scoring systems had been developed to predict short-term mortality in patients with bacteraemia to achieve the improved quality of patient care [4–7]. Regardless of whether the scoring algorithms were adopted in the emergency departments (EDs) [6] or intensive care units [4, 5], the majorities of these scoring systems were derived from clinical information at the time of bacteraemia onset. Although a new tool (i.e., the BLOOMY prediction score) both incorporating clinical data on day 0 and day 3 had been recently developed [7], a scoring system incorporating the dynamic changes in clinical data, which could reflect responses to empirical antimicrobial therapy and early resuscitation, is lacking.

Severe acute respiratory syndrome coronavirus 2 (SARS-CoV-2) was first recognised in December 2019 [8]. On March 11, 2020, the World Health Organization proclaimed the coronavirus disease 2019 (COVID-19) as a worldwide pandemic [9]. The stress caused by the rapid global spread of COVID-19 has been shown to result in the unprecedented consumption of hospital resources [10, 11] and behavior changes in medical teams, such as the delayed diagnosis and treatment of bacteraemia [12]. Additionally, numerous studies detailing the difference of the incidences and causative microorganisms of bloodstream infections before and during the COVID-19 periods have been reported [13–15].

Accurately predicting the length of hospital stay (LOS) enables hospitals to predict the discharge dates of admitted patients and thereby improves the scheduling of elective admissions, reduces bed occupancy variance, and better predicts healthcare costs [16, 17]. Some predictive studies have analyzed the patients who underwent coronary artery bypass grafting [18, 19] and those with critical illnesses [20, 21]. However, the majority of reported predictions have been developed with clinical data gathered at the time of initial hospitalisation [20, 21] or surgery [18, 19]. Research specifically incorporating the changes in clinical data for predicting LOS was lacking among individuals with bacteraemia. Therefore, this study compared the performance of various scoring systems, using clinical information available at the time of bacteraemia onset (day 0), on day 3, and/or changes in variables from day 0 to day 3, in predicting the 30-day mortality and LOS of individuals hospitalised with community-onset bacteraemia.

## Methods

### Study design

This 5-year, multicentre retrospective cohort study was conducted in the EDs of three hospitals in southern Taiwan. The hospitals included one university-affiliated medical centre with 1,200 beds and two teaching hospitals with 460 and 380 beds, respectively. The study enrolled adult patients (age ≥ 18 years) hospitalised with community-onset bacteraemia. The derivation cohort was enrolled from January 2017 to December 2020; the validation cohort was enrolled from January 2021 to December 2021, during the COVID-19 pandemic in Taiwan. The primary and secondary outcomes were the crude mortality rate within 30 days after bacteraemia onset and the LOS, respectively. The scoring systems were established using a joint approach both by conventional regression models and machine learning (ML) methods. The study followed the recommendations of the Strengthening the Reporting of Observational Studies in Epidemiology Initiative.

### Patient selections

During the study period, the results of blood cultures sampled from ED patients were screened for bacterial growth using the electronic medical charts. The inclusion criteria were adults with bacterial growth on blood cultures. For patients with multiple bacteraemic episodes, only the first episode was included. First, this study excluded patients with contaminated blood cultures or bacteraemia diagnosed prior to the ED visits to identify individuals with community-onset bacteraemia. In addition, the study excluded non-hospitalised individuals and those with undetermined mortality or LOS prior to the study endpoint (such as those who had been hospitalised less than 30 days and did not revisit the study hospital). The remaining patients were deemed eligible for study.

### Data collection

**Fig. 1:**
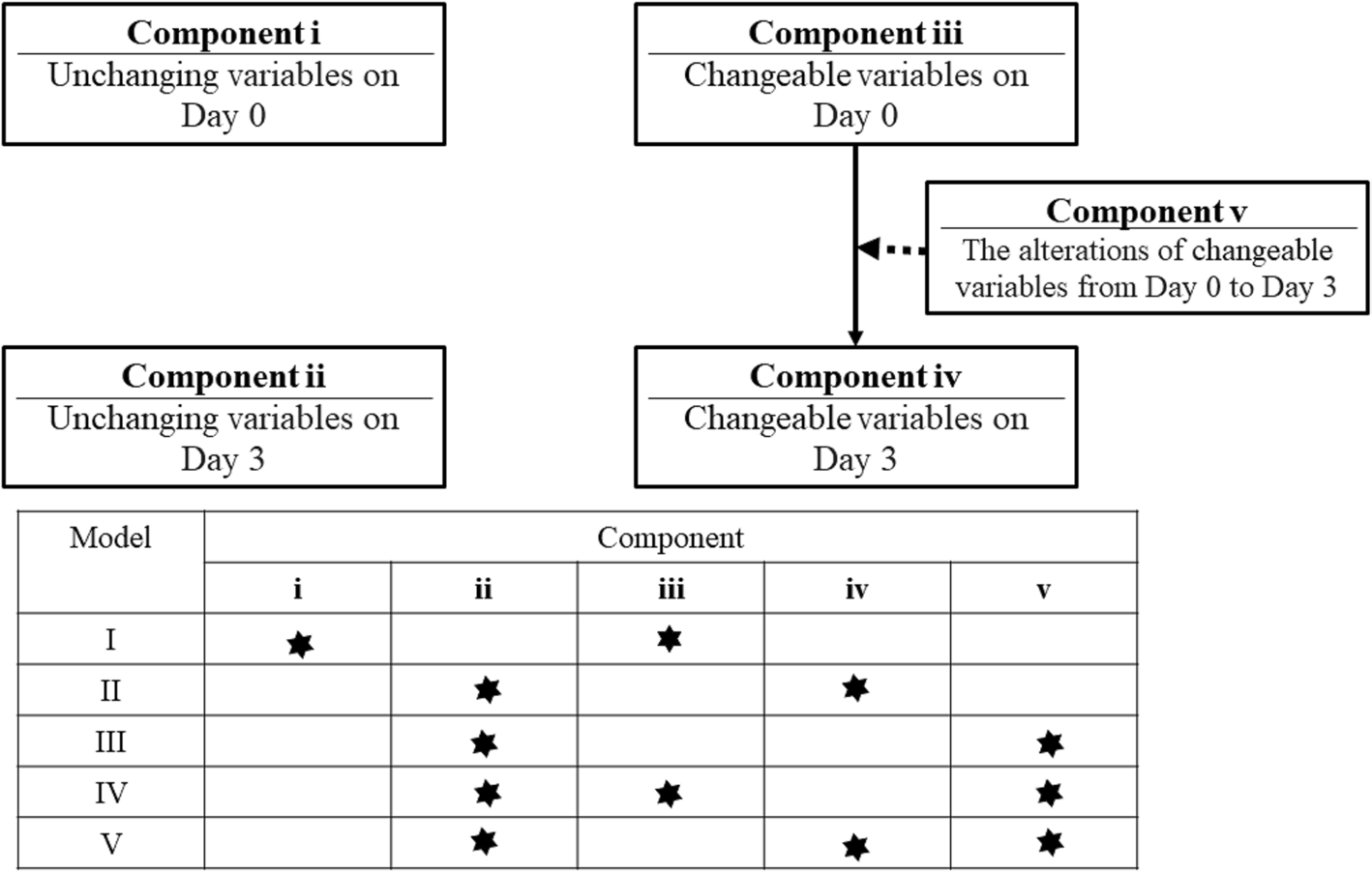
Definition of components and models*. *Day 0 indicates onset of bacteraemia

A predetermined record form was adopted to capture the patient demographic and clinical characteristics of bacteraemia. All information was independently gathered by a board-certified ED physician and an infectious disease physician who were both trained in medical chart reviews; the physicians were blinded to the aim and hypotheses of the present study, and any recording discrepancies were resolved through discussion between the authors. For comprehensive analyses, the clinical data obtained from medical charts were grouped into the following four components: i (unchanging variables on day 0), ii (unchanging variables on day 3), iii (changeable variables on day 0), and iv (changeable variables on day 3). The variables grouped in these components are listed in Supplemental Table 1. Furthermore, component v included alterations in the changeable variables from day 0 to day 3; the alteration descriptions are listed in Supplemental Table 2. The components of Models I, II, III, IV, and V are presented in Fig. 1.

**Table 1:** Clinical manifestations and outcomes in the derivation (non-COVID-19) and validation (COVID-19) cohorts

Variable	Patient number (%)		*P* value
	Derivation *n* = 3639	Validation *n* = 834	
Patient demographic			
Age ≥ 65 years	2181 (59.9)	477 (57.2)	0.15
Gender, male	1892 (52.0)	455 (54.6)	0.18
**Bed-ridden status**	**549 (15.1)**	**100 (12.0)**	**0.02**
Nursing-home resident	215 (5.9)	38 (4.6)	0.13
**Body mass index, mean ± SD**	**22.9 ± 4.9**	**23.7 ± 5.0**	** < 0.001**
Previous events within 4 weeks before bacteraemia onset			
**Hospitalisation**	**762 (20.9)**	**142 (17.0)**	**0.01**
**Chemotherapy**	**265 (7.3)**	**104 (12.5)**	** < 0.001**
Surgery	171 (4.7)	45 (5.4)	0.40
**Invasive procedure**	**101 (2.8)**	**7 (0.8)**	**0.001**
Immunotherapy	43 (1.2)	8 (1.0)	0.59
**Pitt bacteraemia score, median (IQR)**	**1 (0 – 3)**	**2 (0 – 4)**	** < 0.001**
Major bacteraemia source			
Urinary tract	1134 (31.2)	261 (31.3)	0.94
Low respiratory tract	682 (18.7)	148 (17.7)	0.51
Intra-abdominal	441 (12.1)	102 (12.2)	0.93
Skin and soft-tissue	418 (11.5)	84 (10.1)	0.24
Biliary tract	340 (9.3)	87 (10.4)	0.34
**Liver abscess**	**161 (4.4)**	**61 (7.3)**	**0.001**
Polymicrobial bacteraemia	353 (9.7)	65 (7.8)	0.06
**Complicated bacteraemia**	**1033 (28.4)**	**296 (35.5)**	** < 0.001**
Major causative microorganism			
***Escherichia coli***	**1363 (37.5)**	**241 (28.9)**	** < 0.001**
***Klebsiella* species**	**716 (19.7)**	**343 (41.1)**	** < 0.001**
***Streptococcus* species**	**511 (14.0)**	**69 (8.3)**	** < 0.001**
*Staphylococcus aureus*	486 (13.4)	114 (13.7)	0.81
Anaerobes	134 (3.7)	35 (4.2)	0.48
***Pseudomonas* species**	**133 (3.7)**	**19 (2.3)**	**0.048**
Enterococcus species	111 (3.1)	28 (3.4)	0.65
Fatal comorbidity (McCabe classification)	955 (26.2)	215 (25.8)	0.78
Major comorbidity			
Hypertension	1768 (48.6)	386 (46.3)	0.23
**Diabetes mellitus**	**1386 (38.1)**	**357 (42.8)**	**0.01**
Hemato-oncology	1077 (29.6)	274 (32.9)	0.07
Neurological disease	862 (23.7)	188 (22.5)	0.48
**Chronic kidney disease**	**702 (19.3)**	**210 (25.2)**	** < 0.001**
Liver cirrhosis	449 (12.3)	100 (12.0)	0.78
Laboratory data at bacteraemia onset, mean ± SD			
Leukocyte (10^3^/mm^3^)	13.0 ± 11.2	12.7 ± 8.1	0.38
Absolute neutrophile count (10^3^/mm^3^)	11.4 ± 12.3	11.1 ± 7.7	0.46
Hemoglobin (g/dL)	11.8 ± 6.1	11.3 ± 3.3	0.06
Thrombocyte (10^3^/mm^3^)	191.9 ± 115.6	189.4 ± 151.3	0.58
**Blood urea nitrogen (mg/dL)**	**33.8 ± 29.4**	**47.0 ± 36.5**	** < 0.001**
Serum creatinine (mg/dL)	2.0 ± 4.8	2.0 ± 2.6	1.00
Outcome			
**Length of hospital stay, median (IQR)**	**10 (6 – 18)**	**9 (5 – 16)**	**0.002**
Crude mortality rate			
**3-day**	**160 (4.4)**	**96 (11.5)**	** < 0.001**
**30-day**	**602 (16.5)**	**182 (21.8)**	** < 0.001**

*ED* Emergency department, *IQR* Interquartile range

^*^Boldface indicates statistical significance with a *P* value of < 0.05

**Table 2:** The area under ROC of the ML or logistic regression methods in predicting 30-day mortality*

	Derivation cohort**					Validation cohort ***				
	Model I	Model II	Model III	Model IV	Model V	Model I	Model II	Model III	Model IV	Model V
Logistic regression	0.844	0.906	0.880	**0.916**	0.878	0.831	0.8988	0.882	**0.899**	0.885
Random forests	0.707	0.796	0.901	0.985	**0.986**	0.667	0.673	**0.884**	0.718	0.715
SVM	0.699	0.774	0931	**0.969**	0.968	0.662	0.668	0.706	**0.718**	0.707
XGBoost	0.930	0.962	**0.998**	0.973	0.973	0.723	0.734	0.737	**0.745**	0.740
Gradient boost	0.838	0.896	0.982	**0.996**	0.971	0.733	0.722	0.723	**0.744**	0.723
Light GBM	0.712	0.830	0.956	**1.000**	**1.000**	0.695	0.724	0.715	**0.727**	0.708

*ML* Machine learning, *ROC* Receiver operating characteristic, *SVM* Support vector machines, *XGBoost* Extreme gradient boosting, *Light GBM* Light gradient boosting machine

^*^Boldface indicates the highest area under ROC in the derivation and validation cohorts, respectively

^**^Model I was established in 3639 patients and other models in 3479

^*^** Model I was validated in 834 patients and other models in 738

### Definitions

Bacteraemia, the presence of bacteria in the bloodstream, is generally diagnosed with blood cultures after the exclusion of sample contamination. As previously defined [22], community-onset bacteraemia indicated that the episode first identified <48 hours following ED arrival, which included healthcare facility- and community-associated bacteraemia. According to the previous criteria [23], blood cultures with the growth of potentially contaminating pathogens, such as coagulase-negative staphylococci (CoNS), micrococci, *Bacillus* species, *Propionibacterium* species, and Gram-positive bacilli, are considered to be contaminated. The isolation of more than one microbial species from a single bacteraemia episode was classified as polymicrobial bacteraemia. According to the international guideline of the Surviving Sepsis Campaign [24], complicated bacteraemia was defined if a patient fits one of the following criteria: (1) the presence of endocarditis, (2) infections of implanted prostheses, (3) bacterial growth from follow-up blood cultures taken 2-4 days after the initial set, (4) no defervescence at 72 hours after the initiation of appropriate antibiotic treatment, and (5) the presence of metastatic infections.

The Pitt bacteraemia score (PBS) was employed to assess the severity of illness; the score components are vital signs, mental status, use of vasopressor agents, receipt of mechanical ventilation, and cardiac arrest [25]. The comorbid severity was assessed by a previously established classification (McCabe classification) [26]. The overall length of the hospitalisation and ED stay was measured as the LOS. Crude mortality was equated with death from all causes.

### Sampling of blood cultures and microbiological methods

Blood sampling was performed by nurses or physicians in EDs, and two sets of blood cultures were routinely done from different peripheral veins or arteries with at least 30 minutes between the two samplings. A set of blood cultures is routinely composed of one bottle of aerobic culture and another of anaerobic culture, with approximately 10 mL of blood in each bottle. Immediately, blood cultures were incubated in a BACTEC 9240 instrument (Becton Dickinson Diagnostic Systems, Sparks, MD, USA) for 5 days at 35ºC. Bacteraemic isolates were identified by the matrix-assisted laser desorption ionization time-of-flight mass spectrometry.

### Machine learning

Five ML methods, in terms of random forest (RF), support vector machine (SVM), extreme gradient boosting (XGBoost), gradient boost, and light gradient boosting machine (Light GBM), were adopted by ML packages (i.e., scikit-learn, XGBoost, and Light GBM) of Python v3.8 for data preprocessing and building supervised learning models. In the data preprocessing, the method of Multivariate Imputation by Chained Equations (MICE) is used to fill in the missing values. Through multiple regressions over random data, samples get closer to the real dataset. In the process of predictive modeling, classification models and regression models were established using the above five ML methods, along with default hyperparameter settings provided by scikit-learn, XGBoost, and light GBM, for predicting 30-day mortality and LOS, respectively. These ML models were implemented in the following processing: creating an estimator, fitting the training set to the estimator, and predicting new values ​​or class labels for the testing samples. Besides, both classification and regression tasks were implemented on Model I -V to compare their performance.

### Statistical analyses

SAS version 9.4 software (SAS Institute, Cary, NC, USA) was used for statistical analyse. To identify the independent predictors of 30-day mortality, all variables identified as having *P* values < .05 by univariate analyses were included in the backward stepwise logistic regression model. This study developed a scoring algorithm consisting of independent predictive variables to predict 30-day mortality. The area under the receiver operating characteristic (ROC) curve was calculated for all MLs and statistical methods to estimate their accuracy in predicting 30-day mortality.

For predicting LOS, generalized linear models (GLMs) with three different distributions (i.e., normal, negative binomial, and Poisson) were used to recognise the best-fitting model, by the model performance with stepwise selection and an *P* value of <0.05 included variables in the model. The calibration of GLMs was assessed by plotting predicted versus the observed LOS averaged over patients with identical predicted values. The ideal calibration would be indicated by values close to the 45° line on a plot. For the ML models and GLMs, the mean square error (MSE) and root mean square error (RMSE) were employed to evaluate performance in predicting LOS.

## Results

### Patient demographics in the overall cohort

**Fig. 2:**
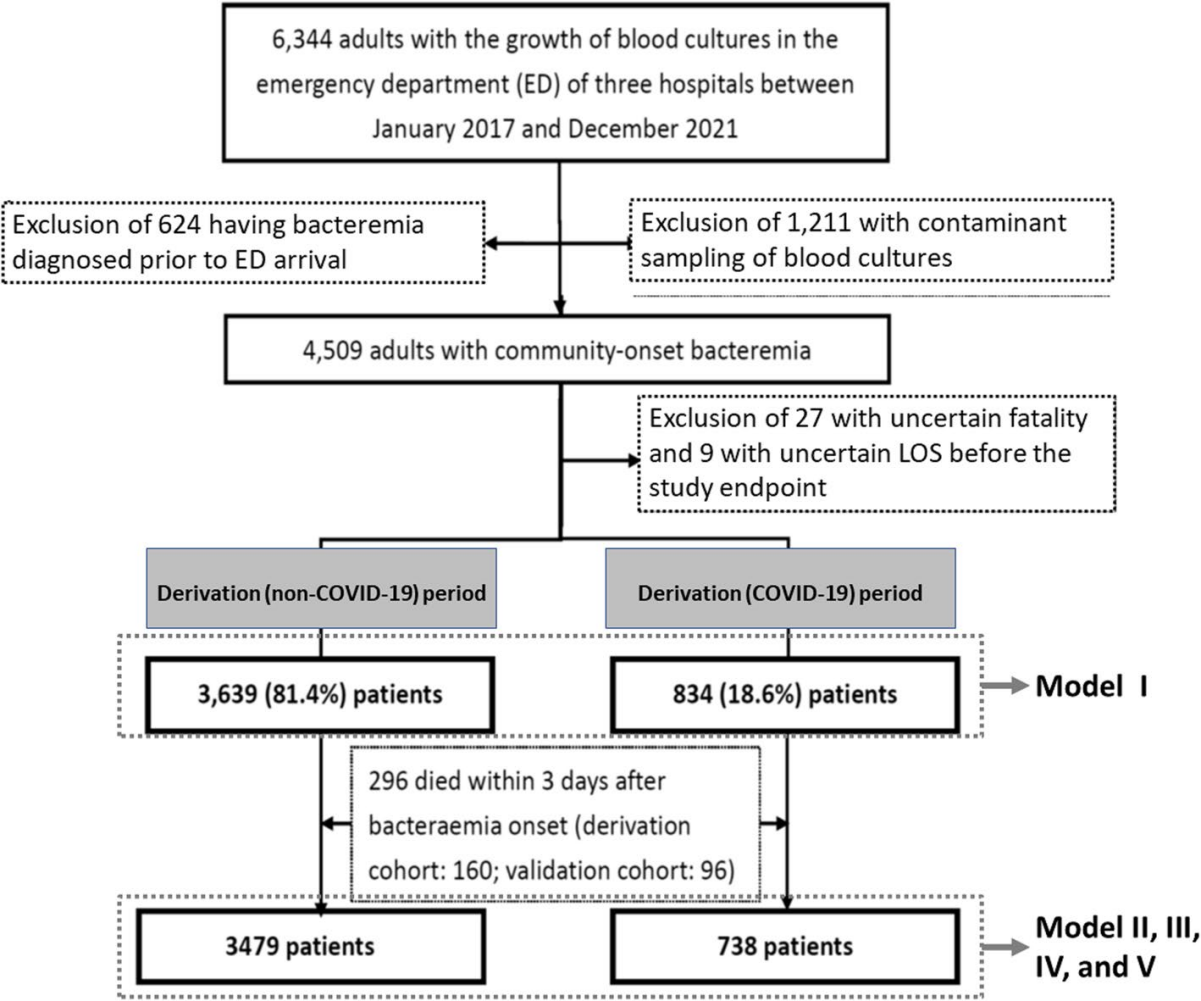
Flowchart of patient selection. *LOS* Length of hospital stay

Of the 6,344 individuals hospitalised with positive blood cultures, 4,473 patients met the study inclusion criteria. The derivation and validation cohorts contained 3,639 (81.4%) and 834 (18.6%) patients, respectively (Fig. 2). Model I was established and validated in the overall cohort (6,344 patients). In this cohort, the median (interquartile range [IQR]) patient age was 69 (57–80) years; 52.4% (2,347 patients) of the patients were male. The LOS after bacteraemia onset ranged from 1 to 293 days, with a median (IQR) of 10 (6–18) days. Of the overall cohort, the patients deemed critically ill (PBS ≥ 4) at the onset of bacteraemia accounted for 23.5% (1,049 patients); the 3-day and 30-day crude mortality rates were 5.7% (256) and 17.5% (784), respectively.

After excluding 296 (6.6%) patients who died within 3 days after bacteraemia onset, 4,217 patients were included in the derivation (3,479 patients, 82.5%) and validation (738 patients, 17.5%) cohorts, respectively, for further analyses using Models II, III, IV, and V. In this cohort, the median (IQR) LOS after bacteraemia onset was 11 (7–19) days, and the 30-day crude mortality rate was 12.5% (527 patients).

### Clinical characteristics and outcomes between the derivation (pre-COVID-19 era) and validation (COVID-19 era) cohorts

Differences between the derivation and validation cohorts with respect to patient demographic characteristics, bacteraemia characteristics, and clinical outcomes are presented in Table 1. Compared with those in the derivation cohort, fewer patients in the validation cohort were bedridden, had previous hospitalisations or invasive procedures, or had the causative microorganisms of *E. coli*, *Streptococcus* species, or *Pseudomonas* species. Conversely, the lower body mass index, the shorter LOS, as well as the few patients with previous chemotherapy, complicated bacteraemia, causative microorganisms of *Klebsiella* species, and comorbidities of diabetes mellitus or chronic kidney diseases were exhibited in the validation cohort. Notably, the validation cohort contained more patients with critical illness at the time of onset and more patients with higher rates of 3-day and 30-day crude mortality compared with patients in the derivation cohort.

### ML or logistic regression in predicting 30-day mortality

The independent predictors of 30-day mortality identified in Models I, II, III, IV, and V were presented in Supplemental Tables 3, 4, 5, 6 and 7. The performance of six methods (i.e., logistic regression, RF, SVM, XGBoost, gradient boosting, and Light GBM) in predicting 30-day mortality as determined using the area under the ROC curve are listed in Table 2. Of the five models established for the derivation cohort, Model IV had the highest area using the logistic regression, SVM, and Light GBM techniques; Model V had the highest area through the RF and gradient boosting techniques; and Model III had the highest area through the XGBoost technique. Furthermore, for the validation cohort, Model IV consistently had the highest area using the logistic regression and five of ML methods (namely the SVM, XGBoost, gradient boosting, and Light GBM techniques).

Regarding the variables incorporated into Model IV, the 10 most powerful predictors of 30-day mortality are identified using logistic regression and the RF, XGBoost, gradient boosting, and Light GBM techniques (Table 3). Of these predictors, the most frequently identified were deteriorated consciousness from day 0 to day 3 (5/50) and deteriorated respiration from day 0 to day 3 (5/50); the other frequently identified variables included stationary shock from day 0 to day 3 (3/50), stationary consciousness from day 0 to day 3 (3/50), improved consciousness from day 0 to day 3 (3/50), and haemoglobin on day 0 (3/50).

**Table 3:** Most ten powerful predictors of 30-day mortality using the ML or logistic regression methods in Model IV

Method	Variables		
	Alteration from Day 0 to day3	Onset of bacteraemia (Day 0)	The characteristic of bacteraemia
Logistic regression	Bacteraemia severity	Bacteraemia severity	Bacteraemia source
	Deteriorated consciousness	Cardiac arrest	Mycotic aneurysm
	Stationary shock	Body temperature ≤ 35.0℃or ≥ 40.0℃	Infective endocarditis
	Deteriorated body temperature		Lower respiratory tract
	Deteriorated respiration		
	Stationary body temperature		
Random forests	Bacteraemia severity	Bacteraemia severity	
	Deteriorated consciousness	Clear consciousness	
	Stationary consciousness	Receipt of mechanical ventilation	
	Improved consciousness	Laboratory data	
	Stationary shock	Hemoglobulin	
	Deteriorated respiration		
	Ventilation dependence		
	Laboratory data		
	Decreased hemoglobulin		
XGBoost	Bacteraemia severity	Bacteraemia severity	Polymicrobial bacteraemia
	Deteriorated consciousness	Comatose consciousness	
	Stationary consciousness	Receipt of mechanical ventilation	
	Improved consciousness	Laboratory data	
	Deteriorated respiration	Neutropenia	
	Stationary shock		
	Laboratory data		
	Deteriorated leukocytosis		
Gradient boost	Bacteraemia severity	Bacteraemia severity	Polymicrobial bacteraemia
	Deteriorated consciousness	Shock	
	Stationary consciousness	Comatose consciousness	
	Appeared arrest	Laboratory data	
	Deteriorated respiration	Hemoglobulin	
	Ventilation dependence	Serum creatinine	
Light GBM	Bacteraemia severity	Laboratory data	Growth number on culture bottle
	Deteriorated consciousness	Hemoglobulin	
	Improved consciousness	Serum creatinine	
	Deteriorated respiration	Blood urea nitrogen	
	Laboratory data		
	Decreased hemoglobulin		
	Elevated serum creatinine		
	Elevated blood urea nitrogen		

*ML* Machine learning;

### ML or GLM methods in predicting the LOS

The calibration curves of GLMs for predicting the LOS for the derivation and validation cohorts are presented in Supplemental Figs. 1 and 2, respectively. The performances of six methods (i.e., logistic regression, RF, SVM, XGBoost, gradient boosting, and Light GBM) in predicting LOS, evaluated using MSE and RMSE, are presented in Table 4. Of the five models constructed for the derivation cohort, Model V had the lowest value for logistic regression and the RF, SVM, and Light GBM techniques; Models II and III had the lowest values for the gradient boost and XGBoost techniques, respectively. Regarding the validation cohort, Model V had the lowest values for the RF, SVM, XGBoost, and gradient boost techniques; Models II and IV had the lowest values for the logistic regression and Light GBM techniques, respectively.

**Table 4:** The mean square error (MSE) and root mean square error (RMSE) of the ML or generalized linear models in predicting the length of hospital stay*

	Derivation cohort**					Validation cohort***				
	Model I	Model II	Model III	Model IV	Model V	Model I	Model II	Model III	Model IV	Model V
	Mean square error									
Logistic regression										
Normal	306.53	273.42	272.78	270.18	**267.61**	210.44	**186.82**	348.22	476.56	354.78
Negative binomial	309.01	283.55	281.38	284.66	**263.31**	220.06	**202.62**	443.52	415.81	589.51
Poisson	317.80	325.14	313.05	308.58	**307.54**	206.37	**206.32**	230.18	364.78	383.93
Random forests	299.94	273.54	**272.94**	283.53	276.21	259.15	295.65	296.78	250.03	**245.99**
SVM	285.25	308.90	309.58	219.29	**218.00**	230.87	263.12	258.98	215.27	**208.54**
XGBoost	517.01	97.54	**28.74**	182.76	180.20	408.19	299.01	350.13	271.60	**271.06**
Gradient boost	225.35	**103.11**	252.33	251.24	249.39	270.53	305.80	245.58	243.00	**238.58**
Light GBM	294.26	313.89	314.07	259.02	**254.83**	247.33	265.15	264.48	**238.41**	238.73
	Root mean square error									
Logistic regression										
Normal	17.51	16.54	16.52	16.44	**16.36**	14.51	**13.67**	18.66	21.83	18.84
Negative binomial	17.58	16.84	16.77	16.87	**16.23**	14.83	**14.23**	21.06	20.39	24.28
Poisson	17.83	18.03	17.69	17.57	**17.54**	14.37	**14.36**	15.17	19.10	19.59
Random forests	17.32	16.54	**16.52**	16.83	16.62	16.10	17.19	17.23	15.81	**15.68**
SVM	16.89	17.58	17.59	14.81	**14.76**	15.19	16.09	16.22	14.67	**14.44**
XGBoost	22.73	9.88	**5.36**	13.52	13.42	20.20	17.29	18.71	16.48	**16.46**
Gradient boost	15.00	**10.20**	15.89	15.85	15.79	16.44	17.49	15.67	15.59	**15.45**
Light GBM	17.15	17.71	17.72	16.09	**15.96**	15.72	16.28	16.26	**15.44**	15.45

*ML* Machine learning, *ROC* Receiver operating characteristic, *SVM* Support vector machines, *XGBoost* Extreme gradient boosting, *Light GBM* Light gradient boosting machine

^*^Boldface indicates the lowest value in the derivation and validation cohorts, respectively

^**^Model I was established in 3639 patients and other models in 3479

^*^** Model I was validated in 834 patients and other models in 738

For the variables integrated into Model V, the 10 most powerful predictors of LOS were identified using GLMs with one of three distributions and the RF, XGBoost, gradient boost, and Light GBM techniques (Table 5). Of these predictors, the most frequently identified variables included deteriorated consciousness from day 0 to day 3 (7/70), a body temperature ≤ 36.0 °C or ≥ 39.0 °C on day 3 (7/70), and a diagnosis of complicated bacteraemia (7/70); other frequently identified variables were blood urea nitrogen on day 3 (5/70), bacteraemia caused by bone and joint infections (5/70), bacteraemia with multiple points of entry (4/70), stationary consciousness from day 0 to day 3 (3/70), ventilation dependence from day 0 to day 3 (3/70), and the receipt of mechanical ventilation on day 3 (3/70).

**Table 5:** Most powerful ten in predicting the length of hospital stay using the ML or generalized linear models in Model V

Method	Variables		
	Alteration from day 0 to day 3	Day 3	Characteristic of bacteraemia or comorbidity
Logistic regression			
Normal	Bacteraemia severity	Bacteraemia severity	Complicated bacteraemia
	Deteriorated consciousness	Body temperature ≤ 36.0℃or ≥ 39.0℃	Bacteraemia source
	Stationary consciousness	Receipt of mechanical ventilation	Multiple port-of-entry
	Improved consciousness	Laboratory data	Urinary tract infection
	Laboratory data	Blood urea nitrogen	
	Elevated blood urea nitrogen		
Negative binomial	Bacteraemia severity	Bacteraemia severity	Complicated bacteraemia
	Deteriorated consciousness	Body temperature ≤ 36.0℃or ≥ 39.0℃	Bacteraemia source
	Stationary consciousness	Laboratory data	Infectious endocarditis
	Improved consciousness	Blood urea nitrogen	Bone and joint infection
	Ventilation dependence		Comorbid malignancy
Poisson	Bacteraemia severity	Bacteraemia severity	Complicated bacteraemia
	Deteriorated consciousness	Body temperature ≤ 36.0℃or ≥ 39.0℃	Bacteraemia source
	Improved consciousness	Laboratory data	Infectious endocarditis
	Ventilation dependence	Blood urea nitrogen	Bone and joint infection
	Stationary respiration		Fatal comorbidity
Random forests	Bacteraemia severity	Bacteraemia severity	Complicated bacteraemia
	Deteriorated consciousness	Body temperature ≤ 36.0℃or ≥ 39.0℃	Causative microorganism of *E. coli*
	Stationary consciousness	Laboratory data	Fatal comorbidity
	Laboratory data	Hemoglobulin	
	Elevated blood urea nitrogen	Serum creatinine	
		Blood urea nitrogen	
XGBoost	Bacteraemia severity	Bacteraemia severity	Complicated bacteraemia
	Deteriorated consciousness	Body temperature ≤ 36.0℃or ≥ 39.0℃	Bacteraemia source
	Ventilation dependence	Receipt of mechanical ventilation	Bone and joint infection
			Multiple port of entry
			Comorbidity type
			Chronic hepatitis
			Coronary artery disease
			Causative microorganism of *E. coli*
Gradient boost	Bacteraemia severity	Bacteraemia severity	Complicated bacteraemia
	Deteriorated consciousness	Body temperature ≤ 36.0℃or ≥ 39.0℃	Bacteraemia source
		Receipt of mechanical ventilation	Infective endocarditis
		Laboratory data	Intraabdominal infection
		Hemoglobulin	Bone and joint infection
		Serum creatinine	Multiple port of entry
Light GBM	Bacteraemia severity	Bacteraemia severity	Complicated bacteraemia
	Deteriorated consciousness	Body temperature ≤ 36.0℃or ≥ 39.0℃	Bacteraemia source
	Laboratory data	Receipt of mechanical ventilation	Infective endocarditis
	Decreased hemoglobulin	Laboratory data	Bone and joint infection
		Hemoglobulin	Multiple port of entry
		Blood urea nitrogen	

*ML* Machine learning, *SVM* Support vector machines, *XGBoost* Extreme gradient boosting, *Light GBM* Light gradient boosting machine

## Discussion

Frontline physicians commonly encounter patients with community-onset bacteraemia, because of its annual incidence of up to 0.15% in the community and the case-fatality rate of highly up to 17% [1] . Therefore, several scoring systems have been developed to predict short-term mortality in patients with bacteraemia to achieve higher quality of care [4–7]. Traditionally, the majorities of these scores were derived from clinical data obtained at the time of bacteraemia onset. Of the models established in the current study, the best performance in predicting 30-day mortality was Model IV, which consisted of unchanging variables on day 3, changeable variables on day 0, and the alterations of changeable variables from day 0 to day 3. Consistent with the BLOOMY score [7], the clinical condition on day 0 and day 3 (as demonstrated in Model IV) had been evidenced as the crucial determinates of short-term fatality. Moreover, similar to updated reports that highlighted the importance of dynamic vital signs and laboratory data in predicting short-term mortality among septic or bacteraemic individuals [27, 28], the changed variables form day 0 to day 3 (as the component in Model IV) were recognised as the powerful determinants of 30-day mortality, in terms of the changes in the conscious level, respiratory condition, and hemodynamic status, which can be recognised as the responses to prompt antimicrobial therapy and early resuscitation.

Accurately predicting LOS at the onset of bacteraemia enables to improve the usage of medical resource and the quality of patient care [16, 17]. In the present study, Model V demonstrated the highest accuracy in predicting LOS by incorporating both unchanging and changeable variables on day 3, along with the changes in changeable variables between day 0 and day 3. In the literature, this is the novel finding emphasized the importance of variables on day 3 and their dynamic changes, incorporated in Model V, as the powerful determinant in predicting LOS, instead of variables at the onset of bacteraemia. Of these determinants, the conscious and respiratory status from day 0 to day 3, blood urea nitrogen and body temperature on day 3, and specific characteristics of bacteraemia (complicated bacteraemia and bacteraemia with multiple ports of entry) were particularly recognised. More importantly, irrespective of whether predicting short-term mortality or LOS, the changes in changeable variables from day 0 to day 3 remained as a crucial determinant in the current study.

Although the SARS-CoV-2 was first detected in late 2019 [8], Taiwan’s response to the COVID-19 pandemic effectively halted the domestic spread of the virus; the government mandated the rapid closure of borders and immediate home quarantines for international arrivals and increased mask manufacturing [29]. These public policies combined with social behaviours initially proved effective in controlling COVID-19, with only 522 recorded cases during 2020 [30]. Unfortunately, SARS-CoV-2 spread rapidly across Taiwan in May 2021, with case numbers rising to 8,924 within one month [30]. Accordingly, the year 2021 was reasonably regarded as a period of the COVID-19 pandemic in the present study.

The global spread of SARS-CoV-2 resulted in the unprecedented demand for hospital resources, mechanical ventilators, beds, personal protective equipment, and medical personnel [10, 11]. Increased demands on healthcare workers could led to the delayed diagnosis and/or treatment of bloodstream infections [12]. Furthermore, the COVID-19 pandemic impacted the incidences and causative microorganisms of bacteraemia [13–15], and the incidence discrepancy and bacteraemia variation resulted from COVID-19-related stress in community individuals and medical teams were highly speculated; this stress in medical teams might agree with a previous investigation indicating a high contamination rate of blood culture in hospitalised patients during the COVID-19 era [31]. Consequently, delayed treatment, bacteraemia variations, and COVID-19-related stresses might result in unfavourable prognoses during the COVID-19 era, as demonstrated in the present study. Consistent with previous studies [14, 15], low incidence of *E. coli* bacteraemia in the COVID-19 era were disclosed in the present study. Dissimilar to previous studies that examined overall types of bacteraemia [14, 15], the altered incidence of *Pseudomonas* and CoNS bacteraemia between the COVID-19 and non-COVID-19 periods was not disclosed because the present study specifically focused on community-onset bacteraemia. In sum, the differences in bacteraemia characteristics and short-term prognoses between the non-COVID-19 and COVID-19 eras was reasonably demonstrated, and thus the COVID-19 era had been appropriately chosen as the validation period in the present study.

Numerous studies have compared the performance of ML models and traditional logistic regression models in predicting mortality [32, 33]. Furthermore, studies have adopted numerous ML methods to predict LOS in the literature [18–21]. For predicting short-term mortality in the non-COVID-19 and COVID-19 eras, Model IV was consistently identified as having the best predictive performance using the majorities of adopted methods in the current study. For predicting LOS in the non-COVID-19 and COVID-19 eras, Model V was consistently identified as having the best predictive performance through the majorities of adopted methods in the present study. Of importance, this study was the first to incorporate changeable data into ML or GLM methods to predict LOS. Consequently, we reasonably demonstrate the crucial role of data that dynamically changed from day 0 to day 3 and the importance of integrating data on day 3 in predicting the LOS and short-term mortality in adults with community-onset bacteraemia.

This study has several possible limitations and multiple strengths. First, the retrospective nature of this study made it prone to the selection and information bias during data collection. To reduce the information bias, all clinical information was randomly and independently retrieved by two physicians who were blind to the hypothesis and they inspected medical records together to solve discrepancies. Second, because of the multicenter design in the present study, the few proportions of patients with uncertain mortality or incomplete clinical information were excluded from analyses, and thereby the selection bias should be negligible. Third, bacteraemia severity and laboratory data had been designed for collection on day 3 because the microbiology reports in blood cultures were generally received by clinicians in the study hospitals on that day; in addition, monitoring of patients from day 0 to day 3 revealed the responses to empirical antimicrobial therapy and early resuscitation. Therefore, the information bias caused by the data missing on day 3 should be trivial in the current study. Finally, because all study hospitals were located in southern Taiwan, the findings in this study may be limited for generalization to other populations, which may have varying causative microorganisms, bacteraemia severity, or severity of comorbidities. However, the present study was the first to provide the external validation of the predicting model on bacteraemia patients in the COVID-19 era.

## Conclusions

The COVID-19 pandemic altered the bacteraemia characteristics and patient demographics among adults with community-onset bacteraemia. Irrespective of the pre-COVID-19 and COVID-19 eras, the importance of dynamic variables changed from day 0 to day 3 (i.e., the indicator in response to empirical antimicrobial therapy and early support care), in predicting the short-term outcomes or LOS was crucially emphasized through the traditional statistic and ML methods in the present study. Accordingly, the principal findings in the current study may contribute to the development of an advanced predictive algorithm and help reduce the disease burden in the nearly future.

### Supplementary Information

**Additional file 1.**

## Data Availability

The datasets used and/or analyzed during the current study available from the corresponding author on reasonable request.
