# Supplementary material for: Predictive models for short-term mortality and length of hospital stay among adults with community-onset bacteraemia before and during the COVID-19 pandemic: application of early data dynamics
Source: BMC Infect Dis. 2023 Sep 15;23:605. doi: 10.1186/s12879-023-08547-8 (PMC10504793; doi:10.1186/s12879-023-08547-8)
Supplement: Supplementary file 1 — Additional file 1. [file 12879_2023_8547_MOESM1_ESM.pdf]

Supplemental Data

Supplemental Figure 1. Calibration curves of the generalized linear model in predicting the length of hospitalisation in the derivation cohort.

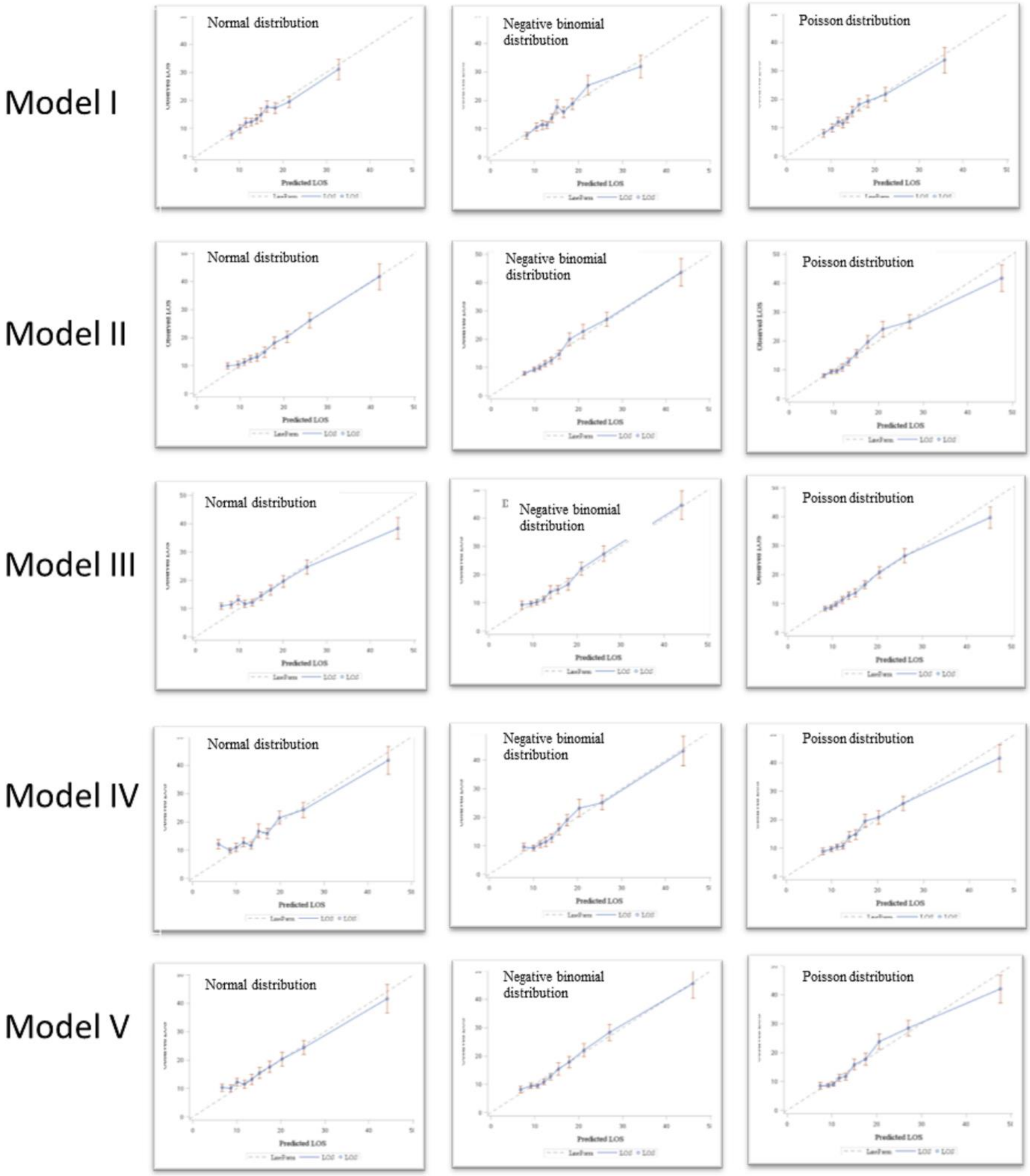

Supplemental Figure 2. Calibration curves of the generalized linear model in predicting the length of hospitalisation in the validation cohort

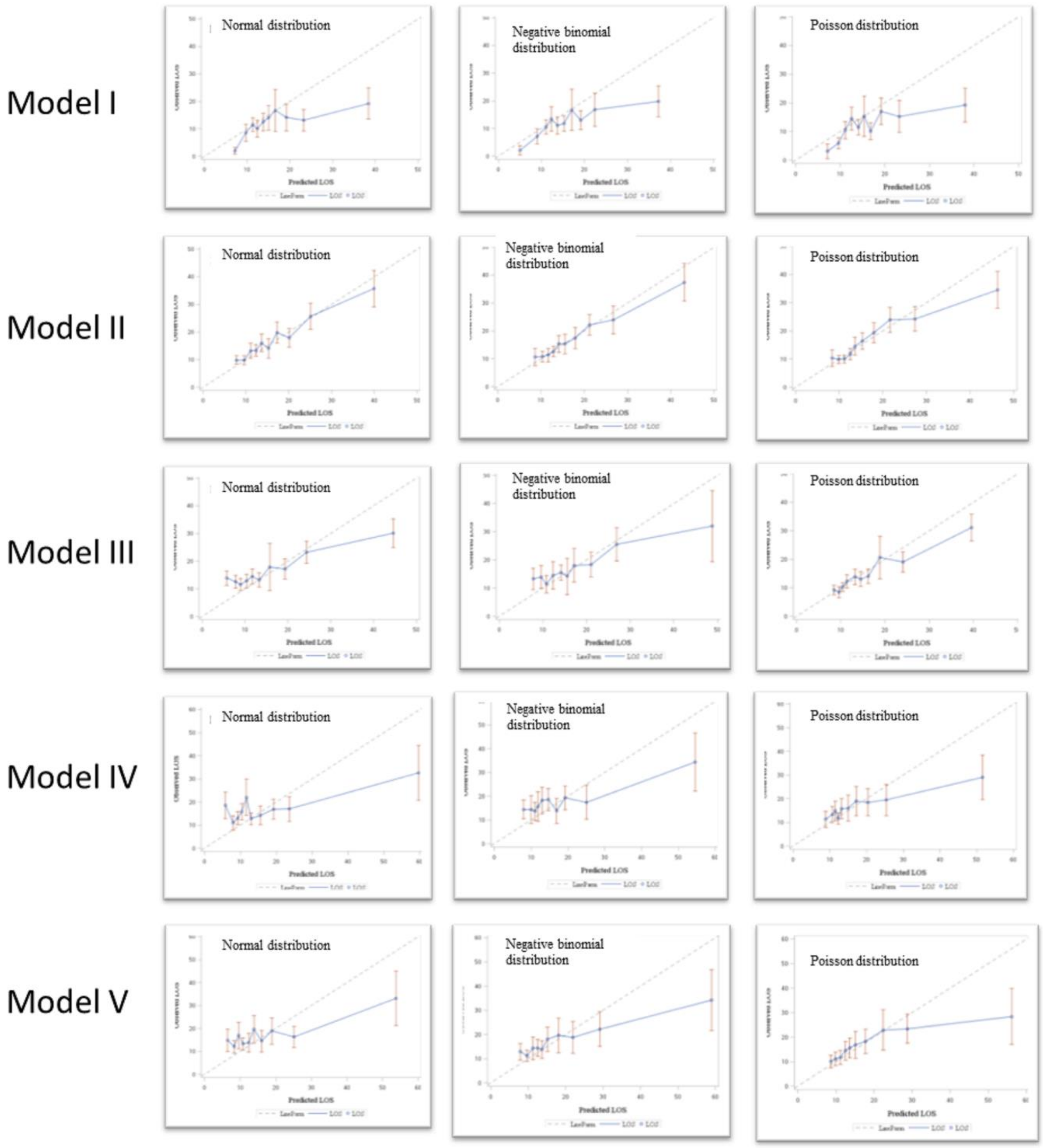

Supplemental Table 1. Details in the component i, ii, iii, and iv.

i	ii	iii	iv
Patient demographic	Patient demographic	Bacteraemia severity on day 0	Bacteraemia severity on day 3
Age	Age	Body temperature	Body temperature
Gender	Gender	Shock	Shock
Body mass index	Body mass index	Receipt of mechanical ventilation	Receipt of mechanical ventilation
Nursing-home resident	Nursing-home resident	Cardiac arrest	Cardiac arrest
Bed-ridden status	Bed-ridden status	Mental status	Mental status
Previous events within 4 weeks before	Previous events within 4 weeks before	Laboratory data on day 0	Laboratory data on day 3
bacteraemia onset	bacteraemia onset	White blood count and classification	White blood count and classification
Hospitalisation	Hospitalisation	Hemoglobulin	Hemoglobulin
Chemotherapy	Chemotherapy	Platelet count	Platelet count
Immunotherapy	Immunotherapy	Blood urea nitrogen	Blood urea nitrogen
Invasive procedure	Invasive procedure	Serum creatinine	Serum creatinine
Surgery	Surgery		
Comorbidity type	Comorbidity type		
Comorbid severity (McCabe	Comorbid severity (McCabe		
classification)	classification)		
Tentative diagnosis for infections	Bacteraemia characteristic		
	Polymicrobial bacteraemia		
	Complicated bacteraemia		
	Source of bacteraemia		

	Causative microorganism<br>Growth number on culture bottle		

Supplemental Table 2. Definitions in the component v.

Body temperature (BT)

	Day 0	Day 3
Improved BT	2	1
	2	0
	1	0
Stationary BT	2	2
	1	1
Deteriorated BT	1	2
	0	1
Reference	0	0

2: ≤35˚C or ≥40˚C
1: 35.1–36.0 or 39.0–39.9˚C
0: 36.1–38.9˚C

Shock

	Day 0	Day 3
Improved hemodynamics	1	0
Stationary shock	1	1
Deteriorated hemodynamics	0	1
Reference	0	0

1: systolic blood pressure < 90 mmHg or the need for inotropic agents to maintain blood pressure
0: not fulfilling above rules

Mechanical ventilation

	Day 0	Day 3
Successful weaning	1	0
Ventilation dependence	1	1
Deteriorated respiration	0	1
Reference	0	0

1: receipt of mechanical ventilation
0: no need of mechanical ventilation

Cardiac arrest

	Day 0	Day 3
Arrest at day 3	Any	1
No arrest at day 3	Any	0
Appeared arrest	0	1
Reference	0	0

1: cardiac arrest
0: no episode of arrest

Mental status

	Day 0	Day 3
Improved consciousness	4	0, 1, or 2
	2	0 or 1
	1	0
Stationary consciousness	4	4
	2	2
	1	1
	0	0
Deteriorated consciousness	0	1, 2, or 4
	1	2 or 4
	2	4
Reference	0	0

4: comatose
2: stuporous
1: disoriented
0: alerted

Leukocytosis

	Day 0	Day 3
Subsided	1	0
Stationary	1	1
Deteriorated	0	1
Reference	0	0

1: blood leucocyte > 9000 /mm3
0: blood leucocyte ≤9000 /mm3

Leukopenia

	Day 0	Day 3
Subsided	1	0
Stationary	1	1
Deteriorated	0	1
Reference	0	0

1: blood leucocyte< 4000 /mm3
0: blood leucocyte ≥4000 /mm3

Bandemia

	Day 0	Day 3
Subsided	1	0
Stationary	1	1
Deteriorated	0	1
Reference	0	0

1: band form ≥ 10% of leucocytes
0: band form < 10% of leucocytes

Neutropenia

	Day 0	Day 3
Subsided	1	0
Stationary	1	1
Deteriorated	0	1
Reference	0	0

1: absolute neutrophile count < 500 /mm3
0: absolute neutrophile count ≥ 500 /mm3

Thrombocytopenia

	Day 0	Day 3
Subsided	1	0
Stationary	1	1
Deteriorated	0	1
Reference	0	0

1: blood platelet < 1500000 /mm3
0: blood platelet ≥ 1500000 /mm3

Supplemental Table 3. Model-I predictors of 30-day mortality in overall 3639 patients (derivation cohort)

Variable	Adjusted OR (95% C.I.)	*P* values
Nursing-home resident	3.77 (2.83 – 5.07)	<0.001
Body mass index <18.5	1.62 (1.20 – 2.17)	0.001
Previous hospitalisation	1.80 (1.29 – 2.51)	<0.001
Fatal comorbidities (McCabe classification)	2.33 (1.73 – 3.13)	<0.001
Comorbid Haemato-oncology	1.92 (1.43 – 2.58)	<0.001
Bacteraemia severity at onset (day 0)		
Shock	7.05 (4.18 – 11.90)	<0.001
Mechanical ventilation	2.88 (2.09 – 3.99)	<0.001

CI = confidence interval; OR = odds ratio.

Supplemental Table 4. Model-II predictors of 30-day mortality in 3479 patients with survival ≥3 days (derivation cohort).

Variable	Adjusted OR (95% C.I.)	*P* values
Fatal comorbidities (McCabe classification)	2.85 (1.96 – 4.13)	<0.001
Polymicrobial bacteraemia	2.26 (1.50 – 3.41)	<0.001
Complicated bacteraemia	1.91 (1.30 – 2.82)	0.001
Bacteraemia severity on day 3		
Conscious unclear	145.97 (82.76 – 257.44)	<0.001
Body temperature <35C or ≥40C	18.73 (7.83 – 44.81)	<0.001
Shock	3.20 (2.03 – 5.05)	<0.001
Mechanical ventilation	1.59 (1.06 – 2.39)	0.03
Laboratory data on day 3		
Neutropenia	2.52 (1.40 – 4.54)	0.002
Thrombocytopenia	1.85 (1.29 – 2.66)	0.001

CI = confidence interval; OR = odds ratio.

Supplemental Table 5. Model-III predictors of 30-day mortality in 3479 patients with survival ≥3 days (derivation cohort).

Variable	Adjusted OR (95% CI)	*P* value
Patient demographic		
Body mass index <18.5	2.14 (1.54 – 2.96)	<0.001
Nursing-home residence	1.84 (1.14 – 2.96)	0.01
Age ≥ 65 years	1.37 (1.02 – 1.84)	0.04
Previous hospitalisation	1.66 (1.23 – 2.25)	0.001
Source of bacteraemia		
Mycotic aneurysm	8.13 (2.21 – 30.01)	0.002
Infective endocarditis	2.68 (1.42 – 5.04)	0.002
Pneumonia	2.63 (1.84 – 3.75)	<0.001
Intraabdominal infection	1.65 (1.07 – 2.54)	0.02
Urinary tract infection	0.60 (0.40 – 0.92)	0.02
Causative microorganism of anaerobe	2.11 (1.10 – 4.07)	0.03
Fatal comorbidity (McCabe classification)	2.18 (1.56 – 3.03)	<0.001
Comorbidity		
Haemato-oncology	1.94 (1.40 – 2.70)	<0.001
Liver cirrhosis	1.67 (1.13 – 2.47)	0.01
Bacteraemia severity from day 0 to day 3		
Body temperature		
Deteriorated	4.09 (2.82 – 5.94)	<0.001
Stationary	2.69 (1.75 – 4.15)	<0.001
Blood pressure		
Deteriorated hemodynamic	9.55 (1.49 – 61.21)	0.02
Stationary shock	6.65 (4.56 – 9.69)	<0.001
Deteriorated consciousness	8.25 (4.04 – 16.85)	<0.001
Deteriorated respiration	3.47 (1.75 – 6.87)	<0.001
Laboratory data from day 0 to day 3		
Stationary bandemia	1.48(1.02 – 2.14)	0.04
Thrombocytopenia		
Deteriorated	1.99(1.38 – 2.88)	<0.001
Stationary	1.71(1.12 – 2.60)	0.01

CI = confidence interval; OR = odds ratio.

Supplemental Table 6. Model-IV predictors of 30-day mortality in 3479 patients with survival ≥3 days (derivation cohort).

Variable	Adjusted OR (95% CI)	*P* value
Patient demographic		
Body mass index <18.5	2.04 (1.47 – 2.83)	<0.001
Previous hospitalisation	1.64 (1.21 – 2.23)	0.002
Source of bacteraemia		
Mycotic aneurysm	9.20 (2.30 – 36.73)	0.002
Infective endocarditis	2.51 (1.33 – 4.74)	0.005
Pneumonia	2.41 (1.69 – 3.46)	<0.001
Intraabdominal infection	1.76 (1.13 – 2.72)	0.01
Urinary tract infection	0.62 (0.41 – 0.94)	0.03
Causative microorganism of anaerobes	2.13 (1.09 – 4.19)	0.03
Fatal comorbidities (McCabe classification)	2.18 (1.55 – 3.06)	<0.001
Comorbidity		
Haemato-oncology	2.11 (1.51 – 2.95)	<0.001
Liver cirrhosis	1.52 (1.02 – 2.26)	0.04
Bacteraemia severity on day 0		
Cardiac arrest	4.60 (2.13 – 9.93)	<0.001
Body temperature <35C or ≥40C	2.26 (1.38 – 3.70)	0.001
Conscious, unclear	2.11 (1.49 – 2.98)	<0.001
Bacteraemia severity from day 0 to day 3		
Deteriorated consciousness	11.21 (4.06 – 22.84)	<0.001
Stationary shock	5.18 (3.50 – 7.66)	<0.001
Body temperature		
Deteriorated	3.80 (2.62 – 5.52)	<0.001
Stationary	2.25 (1.44 – 3.52)	<0.001
Deteriorated respiration	3.04 (1.52 – 6.09)	0.002
Laboratory data from day 0 to day 3		
Thrombocytopenia		
Stationary	1.77(1.16 – 2.70)	0.009
Deteriorated	1.95(1.34 – 2.83)	0.001

CI = confidence interval; OR = odds ratio.

Supplemental Table 7. Model-V predictors of 30-day mortality in 3479 patients with survival ≥3 days (derivation cohort).

Variable	Adjusted OR (95% CI)	*P* value
Patient demographic		
Nursing-home residence	2.04 (1.25 – 3.34)	0.005
Body mass index <18.5	1.99 (1.42 – 2.78)	<0.001
Previous hospitalisation	1.62 (1.19 – 2.22)	0.003
Complicated bacteraemia	1.48 (1.06 – 2.07)	0.02
Source of bacteraemia		
Pneumonia	2.75 (1.92 – 3.96)	<0.001
Urinary tract infection	0.55 (0.36 – 0.84)	0.006
Infective endocarditis	2.72 (1.45 – 5.12)	0.002
Mycotic aneurysm	8.53 (2.35 – 31.04)	0.001
Causative microorganism of anaerobes	2.11 (1.07 – 4.17)	0.03
Fatal comorbidity (McCabe classification)	2.34 (1.65 – 3.30)	<0.001
Comorbidity		
Haemato-oncology	1.94 (1.38 – 2.72)	<0.001
Liver cirrhosis	1.79 (1.22 – 2.63)	0.003
Bacteraemia severity on day 3		
Body temperature <35C or ≥40C	16.88 (8.25 – 34.55)	<0.001
Bacteraemia severity from day 0 to day 3		
Deteriorated consciousness	16.25 (2.98 – 13.13)	<0.001
Blood pressure		
Deteriorated	6.52 (1.04 – 40.92)	0.046
Stationary shock	5.04 (3.37 – 7.54)	<0.001
Deteriorated respiration	4.04 (2.01 – 8.13)	<0.001
Deteriorated body temperature	2.73 (1.85 – 4.03)	<0.001
Laboratory data from day 0 to day 3		
Thrombocytopenia		
Deteriorated	2.01 (1.38 – 2.94)	<0.001
Stationary	1.74 (1.13 – 2.68)	0.01

CI = confidence interval; OR = odds ratio.
